# Rickettsial Infections among *Ctenocephalides felis* and Host Animals during a Flea-Borne Rickettsioses Outbreak in Orange County, California

**DOI:** 10.1371/journal.pone.0160604

**Published:** 2016-08-18

**Authors:** Alice N. Maina, Carrie Fogarty, Laura Krueger, Kevin R. Macaluso, Antony Odhiambo, Kiet Nguyen, Christina M. Farris, Alison Luce-Fedrow, Stephen Bennett, Ju Jiang, Sokanary Sun, Robert F. Cummings, Allen L. Richards

**Affiliations:** 1 Naval Medical Research Center, Silver Spring, MD, United States of America; 2 Orange County Mosquito and Vector Control District, Garden Grove, CA, United States of America; 3 Louisiana State University, Baton Rouge, LA, United States of America; 4 Shippensburg University, Shippensburg, PA, United States of America; 5 West Valley Mosquito and Vector Control District, Ontario, CA, United States of America; Director of Laboratory Sciences, UNITED STATES

## Abstract

Due to a resurgence of flea-borne rickettsioses in Orange County, California, we investigated the etiologies of rickettsial infections of *Ctenocephalides felis*, the predominant fleas species obtained from opossums (*Didelphis virginiana*) and domestic cats (*Felis catus*), collected from case exposure sites and other areas in Orange County. In addition, we assessed the prevalence of IgG antibodies against spotted fever group (SFGR) and typhus group (TGR) rickettsiae in opossum sera. Of the 597 flea specimens collected from opossums and cats, 37.2% tested positive for *Rickettsia*. PCR and sequencing of rickettsial genes obtained from *C*. *felis* flea DNA preparations revealed the presence of *R*. *typhi* (1.3%), *R*. *felis* (28.0%) and *R*. *felis*-like organisms (7.5%). Sera from opossums contained TGR-specific (40.84%), but not SFGR-specific antibodies. The detection of *R*. *felis* and *R*. *typhi* in the *C*. *felis* fleas in Orange County highlights the potential risk for human infection with either of these pathogens, and underscores the need for further investigations incorporating specimens from humans, animal hosts, and invertebrate vectors in endemic areas. Such studies will be essential for establishing a link in the ongoing flea-borne rickettsioses outbreaks.

## Introduction

Murine typhus (also known as endemic typhus) caused by *Rickettsia typhi* has been endemic in California since at least 1915 when the first case was identified [1]. It was recognized as a major public health concern in both Texas and California in the 1940s. This was followed by a marked decline in the number of reported cases in humans, possibly due to the intensive rat and vector control programs that were instituted [2]. In the U.S., human cases of flea-borne rickettsial diseases have been increasing since the 1990s, occurring primarily in southcentral Texas, Hawaii, and southern California [2–4]. Recently an outbreak of rickettsial disease(s) referred to as flea-borne rickettsioses in areas of California traditionally known to be endemic for murine typhus has been reported. Unfortunately the etiologic agent(s) for this outbreak has not been identified [5]. In California during 2013, 105 cases of flea-borne rickettsioses were reported to the California Department of Public Health (CDPH) and were classified as confirmed (n = 67) or probable (n = 38). Overall, 98% of these cases were reported in Orange and Los Angeles counties [3]. Under Title 17, California Code of Regulations (CCR) §2500, Rocky Mountain spotted fever and other rickettsial diseases (non-Rocky Mountain spotted fever), including typhus and typhus-like illnesses, are classified as reportable communicable diseases which must be reported within seven days of identification [6]. It has been noted that in these flea-borne rickettsioses outbreaks, the classical murine typhus urban transmission cycle involving rats (*Rattus norvegicus* and *Rattus rattus*) and the Oriental rat flea (*Xenopsylla cheopis*) has shifted to a suburban cycle that involves cats (*Felis catus*), Virginia opossums (*Didelphis virginiana*), and cat fleas (*Ctenocephalides felis*) [5,7]. This change in vector species for murine typhus is not unheard of as other flea species, lice, and mites have also been implicated as potential vectors and reservoirs for *R*. *typhi* [8,9].

Another possible etiology for the California flea-borne rickettsioses outbreak could be infections with the flea-borne pathogen, *R*. *felis*, which is associated with the prevalent cat flea [5]. *R*. *felis* was first recognized as a human pathogen and the causative agent for flea-borne spotted fever in 1991 in a patient/case in Texas [10], after its detection in a colonized cat flea in 1990 [11]. *C*. *felis* has historically been the only defined biological vector for *R*. *felis*, where the agent is maintained through transovarial and transstadial transmission [12]. However ticks, mites, mosquitoes, and more than 20 species of fleas have been found to be naturally infected with *R*. *felis* [13], including *X*. *cheopis*, the main vector of *R*. *typhi* [4,14–16]

Both *R*. *typhi* and *R*. *felis* have worldwide distribution [17,18] and their areas of endemicity overlap, especially in urban centers and port cities [4,14]. Co-infections with the two species in single cat and rat fleas has been documented to occur both under both natural [4,19] and experimental conditions [20]. Similarly, a previous study conducted in California has shown this sympatric relationship between *R*. *typhi* and *R*. *felis* in cat fleas collected from opossums and cats [5,21]. As a result of their overlapping endemicity, coupled with the sharing of flea vectors, and the similar presentation of clinical illness in humans [17], the actual estimation of the burden and distribution of murine typhus and flea-borne spotted fever is complex [4].

Epidemiologic studies conducted in California and Texas have showed a positive correlation between flea-borne human typhus cases and the geographic distribution of rickettsia seropositive opossums and rickettsia infected fleas [5,22], but no correlation between rickettsia infected fleas and seropositive opossums [5]. Although flea-borne rickettsial diseases due to *R*. *typhi* and *R*. *felis* are well-described individually, significant gaps exist in our knowledge and understanding of their concurrent epidemiology. Here we report the identification of *R*. *felis*, *R*. *typhi*, *Candidatus* Rickettsia senegalensis, and *Candidatus* Rickettsia asemboensis in *C*. *felis*, as well as high prevalence of antibodies against typhus group rickettsiae (TGR) in opossums during the 2011–2013 flea-borne rickettsioses outbreak in Orange County, CA.

## Material and Methods

The study was conducted utilizing three groups of fleas and blood samples from animals collected as part of the comprehensive flea-borne rickettsioses prevention program in Orange County, California, by the Orange County Mosquito and Vector Control District (OCMVCD): 1) opossums caught in small mammal traps (Tomahawk Live Trap®, Tomahawk, WI) set in response to reports of human cases of flea-borne rickettsioses and pet cats owned by humans diagnosed with flea-borne rickettsioses from 2011–2012, 2), opossums collected by OCMVCD as part of routine flea-borne typhus surveillance, but not geographically associated with cases of flea-borne rickettsioses (collections for 2013), and 3) historical biobanked specimens from the county collected in 1969–1988 from opossums and in the environment using “white sock” method [23]. Opossums were euthanized in a CO_2_ gas chamber according to American Veterinary Medical Association (AVMA) protocols [24]. Blood and flea samples from pet cats were taken by local veterinarians. Blood samples and fleas were collected, processed, and stored at -80°C for testing. Fleas were individually washed in molecular-grade water and mechanically disrupted using disposable pellet pestles (Fisher Scientific) or pipette tips. Genomic DNA was extracted from the fleas using Prepman Ultra sample preparation kits (Applied Biosystems, Foster City, CA) and from the opossum sera using DNeasy blood and tissue kits (QIAGEN, Valencia, CA) according to the manufacturers’ instructions, using a final elution volume of 100 μl. A qPCR assay (Rick17b) that amplifies and detects a 115-bp segment of the 17-kDa antigen gene [25] was used to screen flea specimens for the presence of rickettsial DNA. Flea DNA preparations positive for rickettsiae (Rick17b positive) were subsequently tested using species-specific qPCR assay (RFelB) for *R*. *felis* [26] and confirmed with a second species-specific assay (RFel_Phosp_MB) targeting the membrane phosphatase gene from *R*. *felis* [27]. Samples that tested positive with Rick17b but negative for RfelB were further tested using two qPCR assays namely: the *Ca*. R. asemboensis-specific assay (Rasem) that amplifies and detects a 112-bp of the *ompB* gene [15] and the *R*. *typhi*-specific assay (Rtyph) that targets a 122-bp fragment of the *ompB* gene of *R*. *typhi* [28]. PCR amplification and sequencing of *gltA*, *ompB*, *rrs*, *sca4*, and *R*. *felis* plasmid genes *pRF* and *pRFδ* were attempted on a subset of flea DNA samples as previously described [15]. Sequencing reactions were performed utilizing both DNA strands with Big Dye Terminator v3.1 Ready Reaction Cycle Sequencing Kit (Life Technologies, Foster City, CA), according to the manufacturer’s instructions on an ABI 3500 genetic analyzer (Applied Biosystems). Sequences were assembled using CodonCode Aligner version 5.0.1 (CodonCode Corporation, Centerville, MA) and exported to MEGA version 6 software (CEMI, Tempe, AZ) where they were compared with other historical strains sequences available in GenBank. Evolutionary analyses were conducted in MEGA version 6 based upon single-locus sequence typing of *rrs*, *gltA*, *ompB*, *sca4*, and *R*. *felis* plasmid (*pRF*) genes [29]. Chi-squared tests were used to compare the infection rates across the three time periods using the GraphPad Software Inc. (2013). A *p*-value of 0.05 or less was considered significant.

To assess opossums for evidence of previous infection with rickettsiae, serum samples (*n* = 262) were screened in duplicate for spotted fever group rickettsiae (SFGR)- and typhus group rickettsiae (TGR)-specific immunoglobulin G (IgG) at a dilution of 1:100 using enzyme linked immune-sorbent assays (ELISAs) as previously described by Graf *et al*. [30], except that *R*. *conorii* str. Morocco was used as the SFGR ELISA antigen. An anti-opossum conjugate was made using unlabeled anti-opossum antibody from Bethyl Labs, Inc. (Montgomery, TX) and KPL Sure-Link HRP conjugation kit (Gaithersburg, MD). Positive controls were opossum serum pools positive for *R*. *typhi* and cat serum from cats fed upon by *C*. *felis* infected with *R*. *felis* [31]. To confirm the results of the SFGR and TGR ELISAs, 40 random opossum sera were sent to Naval Medical Research Center, Silver Spring, Maryland for quality control and were retested in parallel using both the in-house conjugate and a commercially available anti-opossum IgG (Alpha Diagnostics Intl. Woodlake Center, San Antonio, TX) and anti-cat IgG (KPL, Gaithersburg, MD) labeled with horseradish peroxidase (HRP). An assessment of cross-reactivity of antibodies against *R*. *felis* was conducted using *R*. *felis* immune sera from two cats and two mice with SFGR- and TGR-ELISAs as described above, except that anti-mouse IgG and anti-cat IgG conjugates were used (KPL). The same sera had previously been shown to be reactive to *R*. *felis* str. LSU by IFA as previously described [32].

GenBank Accession numbers: The sequences for the *Candidatus* Rickettsia senegalensis (previously described as *Rickettsia* sp. PU01-02) obtained from *C*. *felis* have been deposited in the GenBank with accession numbers KT304218, KT304219, KT304220 for *rrs*, *ompB*, and *sca4*, respectively.

Ethical approval: The Orange County Mosquito and Vector Control District (OCMVCD) do not have a formal Institutional Animal Care and Use Committee (IACUC) since it is not considered a research institution. However, it does follow the protocols for animal handling for disease surveillance purposes as outlined by the California Department of Public Health, Vector Borne Diseases Section, and adhered to American Veterinary Medical Association (2013) guidelines for animal euthanasia. Scientific permits were obtained from the California Department of Fish and Wildlife (CDFW) to collect and sample small mammals.

## Results

### Flea results

**Fig 1:**
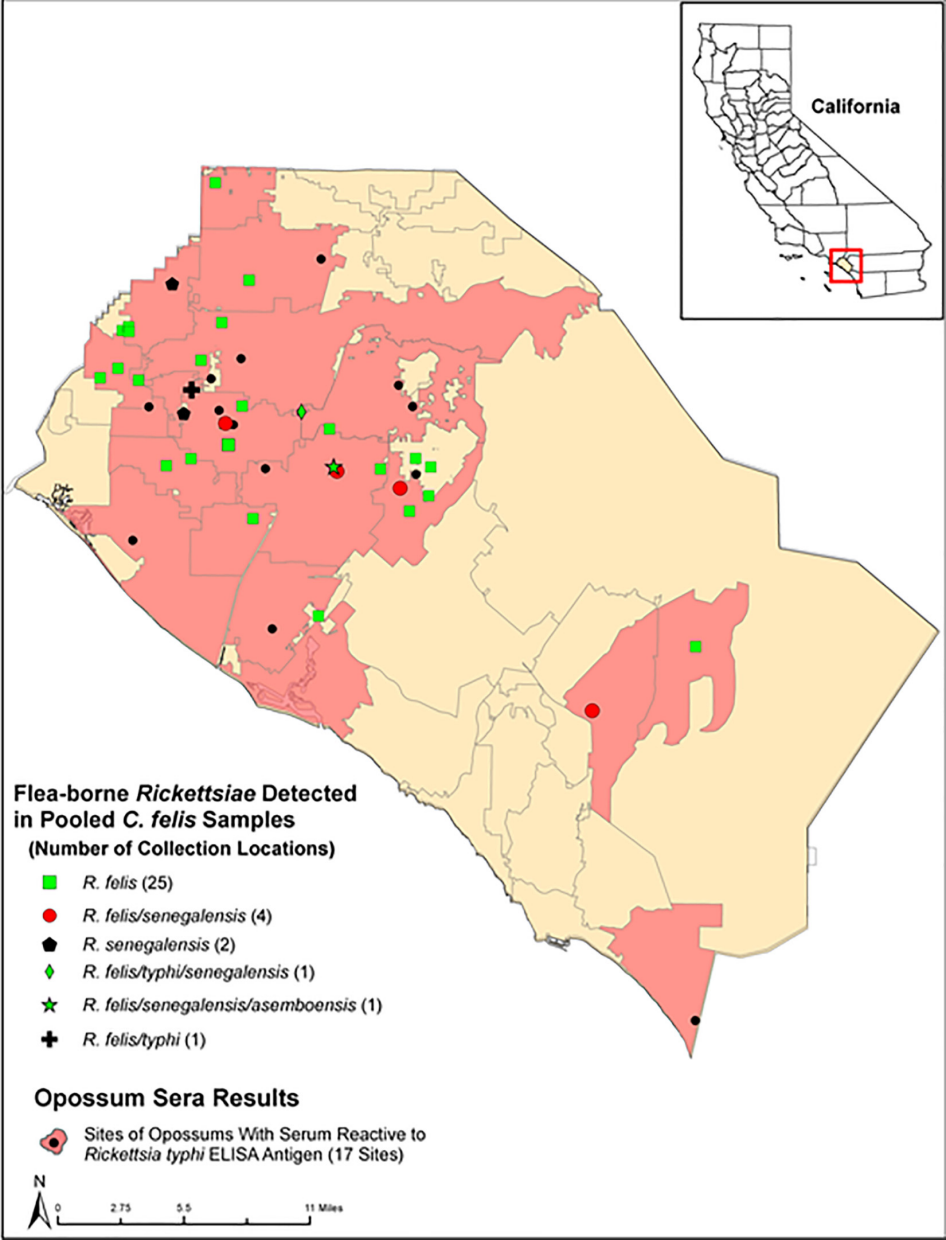
Map showing locations where flea-borne rickettsiae were detected and sites where opossum sera were positive for TGR IgG antibodies in Orange County, California, in 2011–2013.

DNA preparations were obtained from 597 individual *C*. *felis* of which 587 (98.3%) were removed from opossums and 10 (1.75%) were removed from cats. Of the 597 flea DNA extracts, 222 (37.2%) were positive for rickettsial DNA (opossums *n* = 219 of 587 (37.3%) and cats *n* = 3 of 10 (30%)) using the Rick17b qPCR genus-specific assay. The results for the genus-, group- and species-specific qPCR assays are shown in Table 1 and Fig 1. The infection rates were examined by the time periods and the results are summarized in Table 2. No significant difference was found in the rates of infections in the fleas collected in 2011/2012 and 2013 (*p*>0.05). The infection rate for 1969–1988 was higher than 2011/2012 and 2013, but the total numbers of fleas tested for the 1969–1988 period was too small to make a meaningful comparison.

**Table 1:** A summary of qPCR assays results.

qPCR Assay	Specificity	# Pos (%)	Total
**Rick17b**	*Rickettsia* genus	222 (37.2)	597
**Rtyph**	*R*. *typhi*	8 (1.3)	597
**RfelB**	*R*. *felis* & *Ca*. R. senegalensis	212 (35.5)	597
**Rfel_Phos_MB**	*R*. *felis*	169 (28)	597
**Rasem**	*Ca*. R. asemboensis	2 (0.3)	597

**Table 2:** Summary of results by the three surveillance periods.

Groups of fleas tested from	Animal type	qPCR assay	# Flea pos (%)	Total flea
2011–2012	Opossums	Rick17b	141 (39.3)	359
"around case houses and away from reported cases"		Rtyph	5 (1.4)	
		RfelB	134 (37.3)	
		Rfel_Phos_MB	114 (31.8)	
		Rasem	2 (0.6)	
	Cats	Rick17b	3 (30)	10
		Rtyph	0 (0)	
		RfelB	3 (30)	
		Rfel_Phos_MB	3 (30)	
		Rasem	0 (0)	
2013	Opossums	Rick17b	51 (28.8)	177
“throughout the county”		Rtyph	3 (1.7)	
		RfelB	48 (27.1)	
		Rfel_Phos_MB	47 (26.5)	
		Rasem	0 (0)	
1969–1988	Opossums	Rick17b	23 (62.2)	37
“throughout the county”		Rtyph	0 (0)	
		RfelB	23 (62.2)	
		Rfel_Phos_MB	3 (8.1)	
		Rasem	0 (0)	
	No host^#^	Rick17b	4 (28.6)	14
		Rtyph	0 (0)	
		RfelB	4 (28.6)	
		Rfel_Phos_MB	2(14.3)	
		Rasem	0 (0)	

Key

^#^ Fleas were collected in the environment using “white sock” method.

To confirm the identity of rickettsiae identified by the various qPCR assays, PCR amplification and sequencing was performed on either one or a combination of the following genes: *gltA*, *rrs*, *ompB*, *sca4* and/ or *pRF* genes. For RfelB and RFel_Phos_MB positive DNA extracts (n = 11), PCR amplicons were generated and sequences determined for *ompB*. A 1444-bp *ompB* sequence was generated for all 11 DNA preparations, and they were all 100% identical to *R*. *felis* URRWXCal2 (Accession no. CP000053). *R*. *felis* plasmid *pRF* was detected in 3/11 samples and the 402-bp sequence generated for all three was 100% identical to *R*. *felis* URRWXCal2 plasmid *pRF* (CP000054).

**Fig 2:**
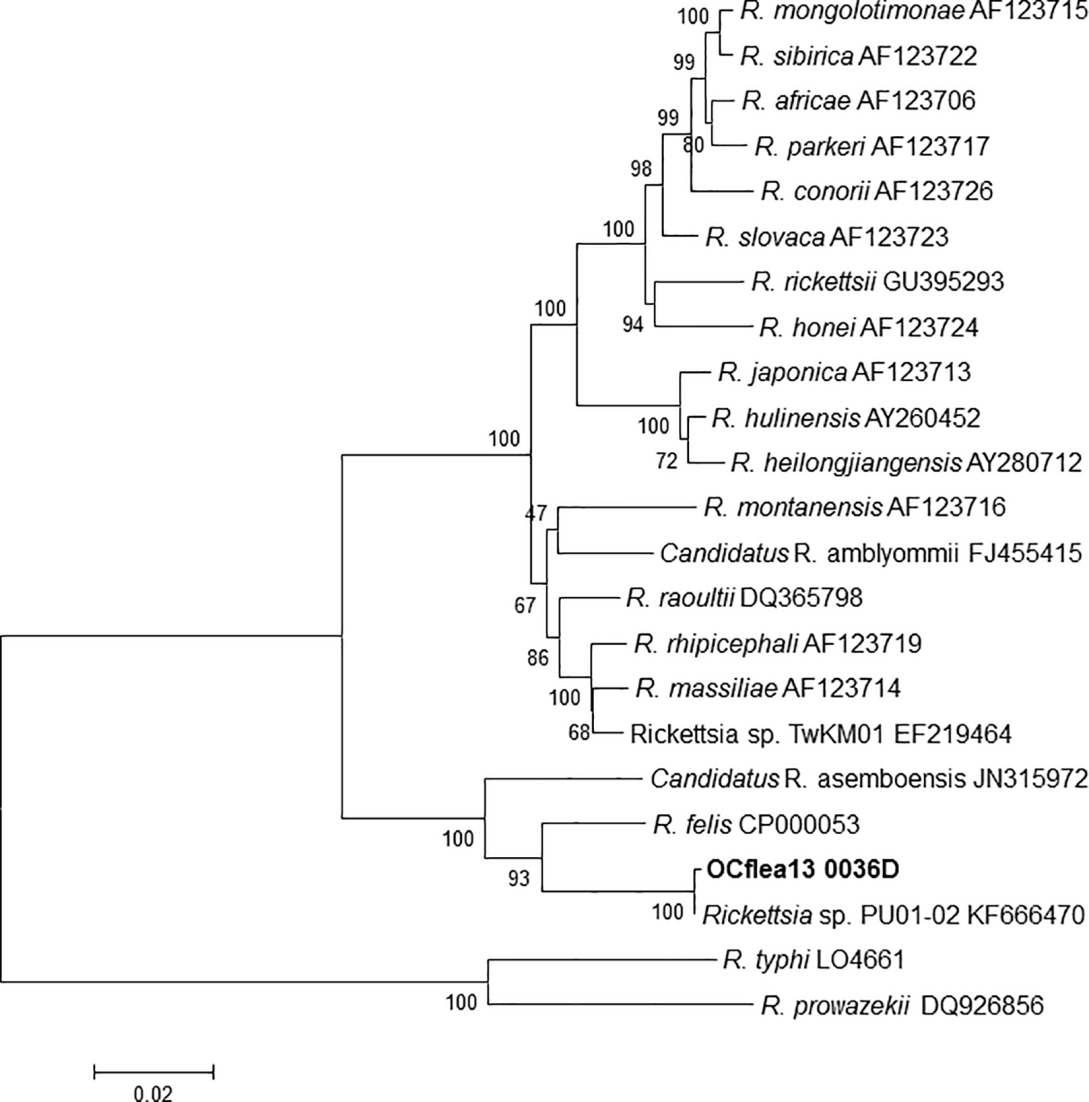
Dendogram of partial sequences of *ompB* gene of rickettsiae from GenBank and OCflea13 0036D detected in *Ctenocephalides felis* obtained from opossums and cats in Orange County, California. The tree was inferred using Maximum Likelihood method involving rickettsiae detected in this study and 22 historical strains provided in GenBank.

A subset of three flea DNA samples that were RfelB positive and /or RFel_Phosp_MB negative were further evaluated to determine their identity. The sequences for *rrs*, *sca4*, and *ompB* genes were determined and compared with those available in GenBank. The three sequences of 1,009-bp fragment of the *rrs* gene were 100% identical with those of *Ca*. R. senegalensis (KF666476). From a 1,046-bp sequence of *sca4* gene, the three DNA preparations had a 100% match to *Ca*. R. senegalensis (KFF666474). From the same 1,046-bp sequence, the similarity of the three preps to that of *R*. *felis* URRWXCal2 (CP000053) was only (98.4%). Phylogenetic relationships inferred from comparison of a 4,292-bp sequence for *ompB* with other *Rickettsia* species in GenBank indicates that *Ca*. R. senegalensis (KF666470) 4,284/4,292 (99.81%) is the closest phylogenetic neighbor to the Orange County molecular isolate (OCFlea 13 0036D) (Fig 2); the closest validated *Rickettsia* species is *R*. *felis* (94.78% match to *R*. *felis* URRWXCal2). A 1431-bp fragment of the *ompB* sequence from the three flea DNA samples was 100% identical to *Rickettsia* sp. DS_018 (KP398500) detected in cat fleas from California. A PCR amplicon for the plasmid *pRF* was not produced for the three samples.

From one of the two flea DNA samples positive by the *R*. *asemboensis*-specific qPCR assay (Rasemb), an amplicon was obtained with the *ompB* PCR. A 1,401-bp *ompB* sequence was determined to be 100% identical to *Rickettsia* sp. DS_006 (KP398499) detected in a cat flea from California and was 99.86% (1399/1401) similar to *Candidatus* R. asemboensis (JN315972). An additional 734-bp sequence of the *ompB* gene was found to be 100% (734/734) identical to *R*. sp. cf9 (DQ379483), 99.86% (733/734) to *Ca*. R. asemboensis (JN315972), 99.73% (732/734) similar to *R*. sp. RF2125 (JX183538), and *R*. sp. R14 (HM370113). Thus, this *R*. *felis* like organism (RFLO) agent was found to be similar to *Ca*. R. asemboensis.

A PCR amplicon (381-bp) for the *gltA* gene was produced in 5 out of the 8 Rtyph positive qPCR assay samples assessed. The *gltA* sequences generated for the five samples assessed were all 100% identical to *R*. *typhi* str. B9991CWPP (CP003398).

Twenty-seven (53%) out of 51 fleas assessed from 1969–88 were positive by Rick17b and RfelB qPCR assays but only 5 (9.8%) were positive by Rfel_Phos_MB qPCR assay. PCR amplification for the *ompB*, *gltA*, and *sca4* genes attempted in 11 DNA extracts from these positive flea DNA preparations yielded no amplicons. A shorter fragment (599-bp) of *ompB* produced amplicons in 4/11 samples. These four samples were 100% identical to *R*. *felis*.

### *Rickettsia typhi* positive opossum sera

A total of 262 opossum sera were assessed for the presence of rickettsia DNA. All the sera were negative for rickettsia DNA using the genus-specific Rick17b qPCR assay. Of the sera tested for the presence of SFGR- and TGR-specific IgG, 107/262 (40.84%) had IgG antibodies reactive against TGR antigens with endpoint titers ranging from 100 to ≥6400 (Fig 1). None of the opossum sera were positive for antibodies against SFGR antigens (S1 Fig).

Positive control serum samples, previously shown to be reactive to the *R*. *felis* str. LSU IFA slides, from two cats fed on by *R*. *felis* str. LSU infected fleas and two mice infected with *R*. *felis* were evaluated by the SFGR- and TGR- specific ELISAs. Serum samples from the two cats and two mice exposed to *R*. *felis*, but not negative control sera, reacted to the SFGR-specific ELISA, but these same samples did not react to the TGR-specific ELISA (S2 Fig). Therefore, the opossum TGR-ELISA positive sera were most likely the result of a previous infection with *R*. *typhi* and not due to a *R*. *felis* infection.

## Discussion

The investigation presented herein follows upon the recent outbreak of flea-borne rickettsial disease in Orange County, California, from 2011–2013 [3,5]. It compares samples collected from opossums caught in small mammal traps around case homes and away from the reported cases during the ecologic investigations of flea-borne rickettsioses (2011–2012), samples from opossums captured throughout the county in 2013, and historical biobanked specimens from the county from 1969–1988. Overall, rickettsial infections were detected in 37.2% of the cat fleas analyzed from opossums and cats. Although *R*. *felis* URRWXCal2 was the predominant rickettsia in the cat fleas (28%), *Ca*. R. senegalensis (7.2%), *Ca*. R. asemboensis (0.3%) and *R*. *typhi* (1.3%) were also detected in the fleas. While flea-borne rickettsioses outbreaks in southern California and Texas have previously been attributed to murine typhus [2,33,34], in relation to *R*. *felis*, *R*. *typhi* was not very common (1.3%) in this investigation in *C*. *felis* fleas removed from opossums and cats. These rates of *R*. *typhi* infections are similar to those reported previously in southern California [5,21].

The RfelB assay [26] previously thought to be specific for *R*. *felis* was found to detect both *R*. *felis* and the newly described *Ca*. R. senegalensis. As such the RfelB assay should no longer be considered a *R*. *felis*-specific assay. However, the recently developed *R*. *felis*-specific assay-RFel_Phosp_MB did not detect *Ca*. R. senegalensis or *Ca*. R. asemboensis, but only detected *R*. *felis* [27]. Thus, we recommend the use of the latter assay in assessing specifically for the presence of *R*. *felis* in arthropod vectors and vertebrate hosts.

PCR and sequencing of either one, or a combination, of *gltA*, *ompB*, *rrs*, *sca4*, and *pRF* genes resulted in the identification of four species within fleas assessed from Orange County. These rickettsiae included: *R*. *typhi*, *R*. *felis*, and the RFLOs *Ca* R. senegalensis, and *Ca*. R. asemboensis. *Candidatus* R. senegalensis was recently isolated in pure culture in XTC-2 cell line from *C*. *felis* fleas removed from cats from Senegal [35]. Like other RFLOs, the pathogenic role of *Ca*. R. senegalensis is unknown. A very close relationship in the *gltA* gene sequence (99.9%) between *Rickettsia* sp. RF31 and *Ca*. R. senegalensis has been noted [35]. The former, *R*. sp. RF31, was first described in *C*. *felis* obtained from the Thai-Myanmar border, however limited gene sequence information was reported [36]. In the United States *R*. sp. RF31 was first reported in cat fleas in South Carolina in 2005 [37], and belonging to this group also is DS-018 (KP398500) detected in California [38]. This report confirms existence of *Ca*. R. senegalensis (or a very closely related agent) in California, United States.

We report in addition to *R*. *typhi*, *R*. *felis*, and *Ca*. R. senegalensis, the presence of *Ca*. R. asemboensis among *C*. *felis* fleas. We speculate that *Rickettsia* sp. DS_006 (KP398499) reported in California [38], and other closely related genotypes reported worldwide [15,26,36,39,40] are the same as *Ca*. R. asemboensis. Within this genogroup, only *Ca*. R. asemboensis has been isolated in cell culture and has the full genome sequence available in GenBank [41]. Recently, a *Ca*. R. asemboensis-genotype was detected in the blood of a monkey (*Macaca fascicularis*) in Malaysia [42]. Although the *Ca*. R. asemboensis genogroup appears to have a wide distribution, it has not been associated with any clinical illness.

During this study, DNA from archived cat flea samples collected from 1969 through 1988 were also extracted and tested. Rickettsia DNA was detected in these samples using the pan-rickettsial qPCR assay targeting the 17kDa (Rick17b) and species-specific assays (RFelB and RFel_Phosp_MB) indicating that *R*. *felis* was circulating in *C*. *felis* collected in Orange County. Unfortunately, it was not possible to produce the longer PCR amplicons necessary for sequencing gene fragments of *ompB*, *ompA*, and *sca4*. This may be due to DNA degradation resulting from prolonged storage. We were unable to confirm the rickettsia present in samples collected in 1969–1988. Comparing results from 2011–2012 and 2013, RFLOs only made up 0–15% of all RfelB positives. It is therefore more likely that a majority of positive fleas contained *R*. *felis* DNA and very little if any RFLOs. Interestingly, we detected *R*. *felis* DNA in fleas collected 1969–1988, suggesting that *R*. *felis* was circulating in the flea population prior to its first description in 1990.

Rickettsial DNA was not found in the blood of opossums in this study, which is consistent with the results from previous investigations in which rickettsial DNA and/or evidence of rickettsemia was not detectable in the blood of opossums [5,43], peridomestic small mammals [33], and the Norway rat (*Rattus norvegicus*) [39]. Additionally, in an experimental study, intraperitoneal inoculation of *Didelphis aurita* (black-eared opossum) with *R*. *felis* did not produce rickettsemia [44]. Although the present study and other previous studies did not find rickettsemia in the opossums’ blood, *R*. *felis* DNA has been detected in tissues from opossums and *Rattus norvegicus* [5,45], while *R*. *typhi* DNA was detected in the spleen of the opossum [46].

Our study reports a high prevalence (40.84%) of antibodies against *R*. *typhi*, however antibodies against SFGR were not detected in these same samples. This is especially intriguing since the opossums were infested with fleas (average flea index of 53.5, range 0–501 fleas per animal), of which 29.3% were infected with the rickettsial pathogens: *R*. *typhi* (TGR) (1.3%) and *R*. *felis* (SFGR) (28%). This observation is in agreement with previous studies, which reported anti-*R*. *typhi* antibody prevalence of 42–71% in opossums trapped within the vicinity of human flea-borne rickettsioses cases in Los Angeles County and Austin, Texas [22,33], and an 11% prevalence in Orange County [7]. However, a recent study conducted in Orange County, and coinciding with a flea-borne rickettsioses outbreak, did not detect any anti-*R*. *typhi* antibodies in the opossums by IFA [5]. Although the classic transmission cycle for murine typhus involves infected rats and rat fleas (*X*. *cheopis*), the latter are rarely reported in these flea-borne rickettsial disease endemic areas [22,39]. While *X*. *cheopis* is known as the biological vector for *R*. *typhi*, our study found no *X*. *cheopis* on opossums and cats, and 1.3% of cat fleas infesting opossums were infected with *R*. *typhi*. These findings align with studies conducted from 1916 through 1948 where an association between flea-borne rickettsial disease with opossums and cat fleas had already been made [47,48]. The previous reports, together with the high seropositivity for *R*. *typhi* antibodies in the opossums suggest that the opossums and cat fleas may play an important role in the epidemiology of murine typhus [8].

Although opossums have been shown to be susceptible to *R*. *felis* infection through experimental studies [44] and natural infection [2], the present study did not detect antibodies against SFGR in the opossums assessed. Contrastingly, high seroprevalence (22%) to anti- *R*. *felis* has been noted in opossum populations in Texas [2], utilizing *R*. *typhi* str. Wilmington and *R*. *felis*-infected cat flea midguts on IFA slides. Boostrom et al. [2] noted that 8% of opossum sera were reactive to *R*. *typhi*, but their IFA could not distinguish *R*. *typhi* from *R*. *felis* in 6 samples, denoting cross-reactivity or dual infections. In the present study, our ELISA, utilizing serum from mice infected with, and cats fed upon by cat fleas infected with, *R*. *felis* str. LSU, reacted with *R*. *conorii* str. Morocco but not with *R*. *typhi* str. Wilmington ELISA antigens. Fang and Raoult [49] found that IgG antibodies against *R*. *felis* cross-reacted more often with antigens from SFGR and less with antigens from TGR using microimmunofluorescence (MIF). Though some studies based only on IFA suggest that flea-borne spotted fever induced antibodies react well to both *R*. *typhi* and *R*. *felis* IFA antigens [10,50], the ELISAs used in this study did not show such crossreactivity.

Our study provides two important findings, first that cat fleas were predominantly infected with *R*. *felis*, but to a lesser extent with *R*. *typhi* and RFLOs. Second, in contrast to the small proportion of *R*. *typhi* infected fleas in relation to *R*. *felis* infected fleas, opossums had high prevalence of anti-*R*. *typhi* IgG antibodies and none to SFGR. The epidemiology of flea-borne rickettsial diseases warrants further investigations to incorporate specimens from humans, animal hosts, and invertebrate vectors in endemic areas to establish a link in the ongoing flea-borne rickettsioses outbreaks.

## Supporting Information

S1 Fig Aphotograph of screening plates showing both positive (dark green) and negative (colorless circles) for *R*. *typhi*- specific IgG antibodies in opossum sera (plates A and B) and negative for *R*. *conorii*-specific IgG antibodies (plates C and D). Note the positive control (red arrows) and the negative controls (blue arrows). Serum from a cat infected with *R*. *felis* was evaluated with the SFGR and TGR ELISAs. Cat serum with anti-*R*. *felis* reacted with *R*. *conorii* antigen of the SFGR ELISA (plate C, red arrows).
(TIF)

S2 Fig Orange County titer data for the opossum sera (n = 23) positive for anti-*R*. *typhi* antibodies during screening (Plates A,B and C). Note that for plate A, the positive control (column 1), 3 negative controls (column 2–4) and cat serum infected with *R*. *felis* was included as a control for *R*. *typhi* antigen (column 5, yellow arrow). The cat serum with anti-*R*. *felis* did not react with *R*. *typhi* antigen of the TGR ELISA (panel yellow arrow).
(TIF)
