# Supplementary figures and images for: Rickettsial Infections among Ctenocephalides felis and Host Animals during a Flea-Borne Rickettsioses Outbreak in Orange County, California
Source: PLoS One. 2016 Aug 18;11(8):e0160604. doi: 10.1371/journal.pone.0160604 (PMC4990410; doi:10.1371/journal.pone.0160604)

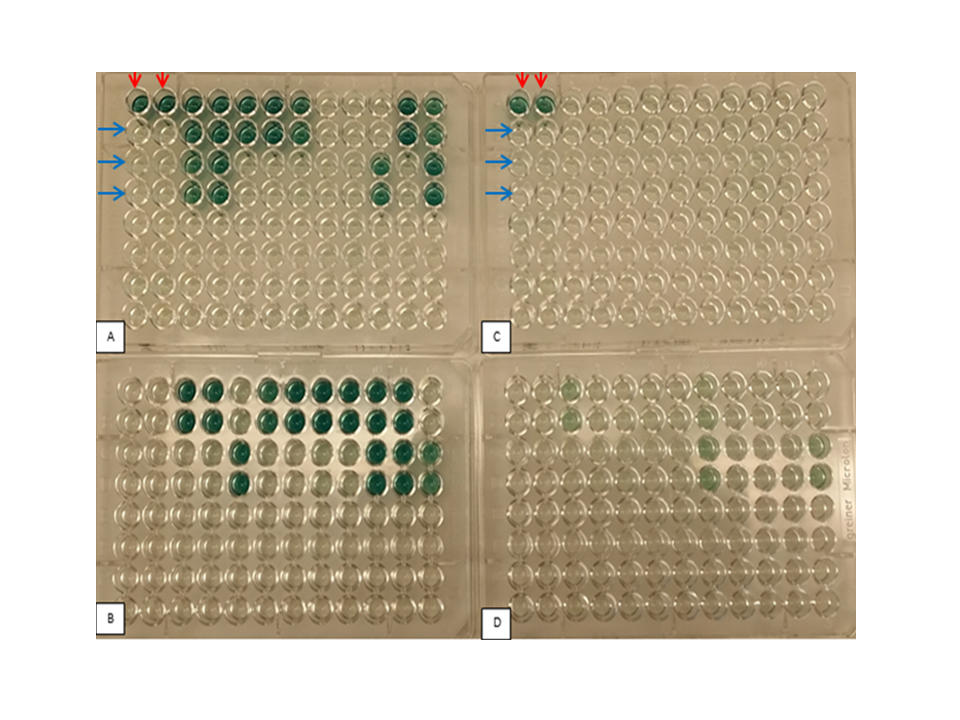

Supplement: S1 Fig — Aphotograph of screening plates showing both positive (dark green) and negative (colorless circles) for R. typhi- specific IgG antibodies in opossum sera (plates A and B) and negative for R. conorii-specific IgG antibodies (plates C and D). Note the positive control (red arrows) and the negative controls (blue arrows). Serum from a cat infected with R. felis was evaluated with the SFGR and TGR ELISAs. Cat serum with anti-R. felis reacted with R. conorii antigen of the SFGR ELISA (plate C, red arrows). (TIF) [file pone.0160604.s001.tif]

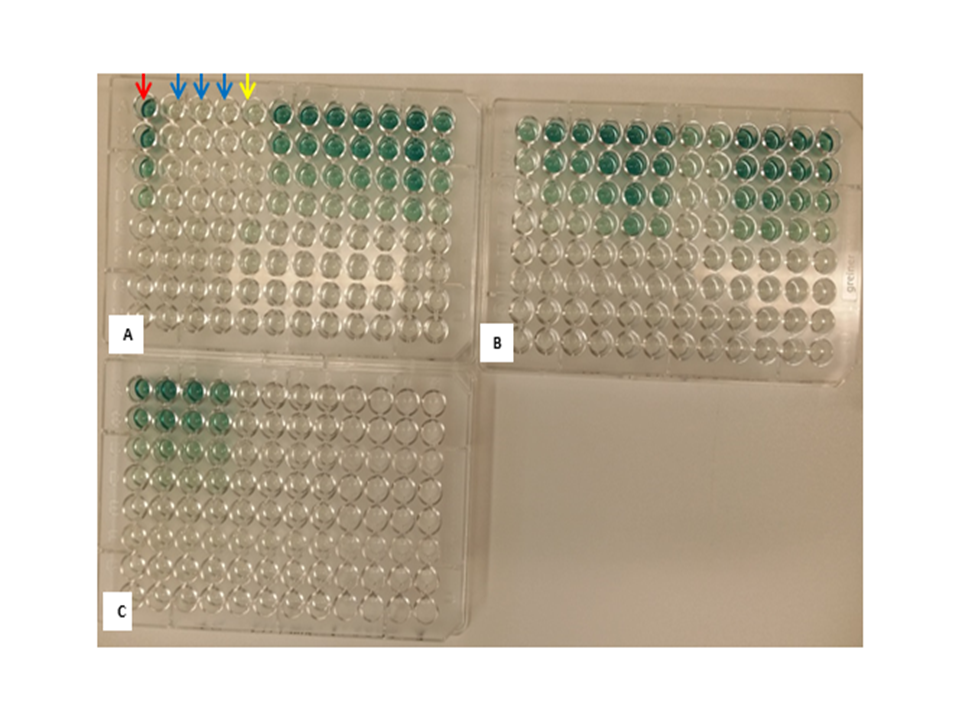

Supplement: S2 Fig — Orange County titer data for the opossum sera (n = 23) positive for anti-R. typhi antibodies during screening (Plates A,B and C). Note that for plate A, the positive control (column 1), 3 negative controls (column 2–4) and cat serum infected with R. felis was included as a control for R. typhi antigen (column 5, yellow arrow). The cat serum with anti-R. felis did not react with R. typhi antigen of the TGR ELISA (panel yellow arrow). (TIF) [file pone.0160604.s002.tif]
